# The analytics–practice gap: why sports data fails to translate into coaching decisions

**DOI:** 10.3389/fspor.2026.1866960

**Published:** 2026-07-07

**Authors:** Jun Woo Kwon

**Affiliations:** Department of Physical Education, Sports Technology Laboratory, Seoul National University, Seoul, Republic of Korea

**Keywords:** coaching practice, decision-making, key performance indicators, performance analysis, sports analytics

## Abstract

The rapid expansion of tracking systems, wearable sensors, video-based analysis platforms, and analytical models has created increasingly data-rich environments in sport. However, the extent to which these developments translate into coaching decisions remains uncertain. In applied settings, decisions about training design, tactical preparation, player selection, workload management, and in-game adjustment often continue to rely heavily on experiential knowledge, contextual judgement, and established practice. This paper examines this disconnect, conceptualized as the analytics–practice gap, and argues that the gap is not primarily caused by insufficient data, but by the limited actionability, contextualization, integration, and usability of many analytical outputs. Performance indicators and advanced metrics may describe what occurred, but they do not automatically clarify what coaches should do next. Addressing this gap requires a shift from data-first to decision-first analytics. This involves defining the coaching problem before selecting metrics, integrating contextual information, synthesizing technical, tactical, and physical data, and communicating outputs in ways that support practical intervention. Ultimately, the value of sports analytics depends less on the capacity to generate additional data and more on the ability to connect existing information to actionable changes in training design, tactical planning, player management, and performance preparation.

## 1 Introduction: a data-rich but decision-poor landscape

Over the past two decades, sports performance analysis has expanded substantially. This growth has been driven by developments in tracking technologies, wearable sensors, automated event data, video-based analysis platforms, and computational modelling. In elite and competitive team-sport settings, coaches and analysts now have access to increasingly detailed information about technical actions, tactical behaviors, physical outputs, and match contexts. Contemporary approaches can integrate multiple data streams to describe performance (1, 2), while advances in match analysis and tactical analytics have further shaped how performance is interpreted (3). These developments have contributed to the view that sport is becoming increasingly data-driven.

In this paper, performance analysis and sports analytics are treated as related but distinct practices. Performance analysis refers to the broader applied process through which performance information is observed, coded, interpreted, and communicated to support coaching feedback. In applied settings, this process does not depend only on numerical data. It also relies heavily on video, notational analysis, coach–analyst dialogue, and contextual interpretation, particularly when feedback is used to inform training or match preparation (4, 5). Sports analytics is used more narrowly to describe data-driven, statistical, computational, model-based, or spatiotemporal approaches that support interpretation and decision-making (1, 2, 6). The two areas overlap in practice, but they are not identical. This distinction is important because the analytics–practice gap is not only a problem of data availability. It is also a problem of how performance information, whether derived from video, indicators, tracking systems, or models, is translated into coaching action.

However, greater access to data has not necessarily produced a corresponding improvement in coaching decision-making. Coaches still operate in environments shaped by uncertainty, time pressure, experiential knowledge, and established routines (7). In this context, the practical value of analysis depends not only on whether information is available, but on whether it can be interpreted in relation to the decisions coaches actually need to make. Recent umbrella-review evidence in team ball sports supports this concern. Sarmento et al. showed that match analysis research has expanded considerably (8), but remains dominated by individual-level analyses and still requires more integrated approaches that consider physical, technical, tactical, and psychosocial dimensions of performance. They also noted that match context, including match status, location, period, and quality of opposition, requires deeper consideration.

This issue is also evident in the use of performance indicators. Key performance indicators (KPIs) are widely used in performance analysis to quantify observable aspects of play (9, 10). Yet their interpretation is rarely straightforward. Common measures such as ball possession or passing accuracy may appear to describe performance quality, but their meaning can change according to match status, opposition level, playing style, and tactical intention (11). Morgulev and Lebed similarly argued that performance analysis and sports analytics often prioritize variables that are easily counted and widely accessible (6), rather than variables that necessarily explain the dynamic interactions through which performance emerges. In such cases, more data do not automatically produce better understanding.

Advanced analytical methods raise a similar issue. Herold et al. showed that tracking-derived football metrics can be used within video feedback, but also that analytically meaningful information may have limited impact when it is not sufficiently embedded in coaching explanation, training design, and behavioral change (12).

These observations point to a broader issue. The challenge is not simply the lack of data, nor is it solved by adding more complex models. The central difficulty lies in translating available information into actionable coaching decisions. This raises the guiding question of this paper: why does increasing data availability not consistently lead to improved decision-making in sport?

## 2 The analytics–practice gap

The rapid expansion of performance data in sport has not been matched by an equivalent transformation in coaching practice. Analytical tools and monitoring systems have become more sophisticated, but their integration into day-to-day coaching decisions remains uneven. This discrepancy can be understood as the analytics–practice gap: the disconnect between producing performance data and applying it meaningfully in coaching. In applied settings, the gap may appear in simple but important ways. A report may show that high-intensity running declines in the final stages of a match, but it may not clarify whether the coach should adjust conditioning work, substitution timing, or pressing strategy. A passing-network analysis may describe how a team circulates the ball, but still leave open how build-up play should be trained. A workload alert may signal increased external load, but remain difficult to interpret without information about player readiness, tactical role, injury history, and upcoming match demands.

This gap does not arise from a lack of analytical capability. Contemporary sport environments are equipped with monitoring systems capable of tracking technical actions, tactical patterns, and physiological load with high resolution (13). Advances in performance analysis have also enabled detailed descriptions of game dynamics and player behavior, supported by large datasets and increasingly complex models (2). From a technical standpoint, the capacity to generate information is not the main limitation.

The more difficult problem is translation. Research embedded within high-performance sport has shown that the transfer of knowledge from scientific analysis to applied settings is often constrained by contextual and organizational factors (7). Coaches work under time pressure and uncertainty, and they often need to make decisions before the evidence is complete. Under these conditions, the practical value of data depends less on its volume or complexity than on whether it is relevant, understandable, and usable. A metric becomes useful when it helps answer a coaching question: whether to modify training intensity, change a tactical emphasis, adjust player rotation, or alter feedback before the next match.

A central problem is that information is often collected because it can be captured, rather than because it is directly linked to a coaching decision. Tracking systems, wearable sensors, video platforms, and event-data providers can produce large quantities of information. Yet the availability of a variable does not make it actionable. Morgulev and Lebed make a similar point in their theoretical-methodological discussion of performance analysis and sports analytics, arguing that the field may prioritize variables that are easily counted and widely accessible rather than variables that explain the dynamic interactions of performance (6). This can create a data-rich but decision-poor workflow in which metrics are collected, visualized, and discussed, but remain weakly connected to training design or match preparation.

The same problem can occur with more advanced analytics. Relevant methods may include tracking-derived tactical metrics, passing-network analysis, possession-value models, expected-goals models, clustering of playing styles, and predictive workload models. These approaches can provide richer information than isolated descriptive indicators, but they still require coaching interpretation. Recent work on off-the-ball behaviors in football illustrates how tactical behaviors can be made more explicit through a structured taxonomic process that specifies the function of the behavior, the unit of analysis, the performance dimension, and the behavioral type (14). This type of framework shows that the value of network- or tracking-derived metrics depends not only on calculation, but also on whether the behavior being measured is clearly defined and interpretable for subsequent coaching discussion. For example, a possession-value model may identify where a team creates or loses attacking value, but the applied question is whether this should change spacing principles, passing options, training constraints, or opposition-specific preparation.

Applied research illustrates both the promise and limitation of this translation process. Herold et al. used positional tracking-derived measures of passing effectiveness in a video-feedback intervention with professional football players (12), yet the intervention did not produce significant improvements in the main tracking-derived outcomes. This example shows that analytics can enter applied practice while still having limited impact if feedback is not sufficiently connected to coaching explanation, field-based training tasks, and behavioral change.

**Figure 1:**
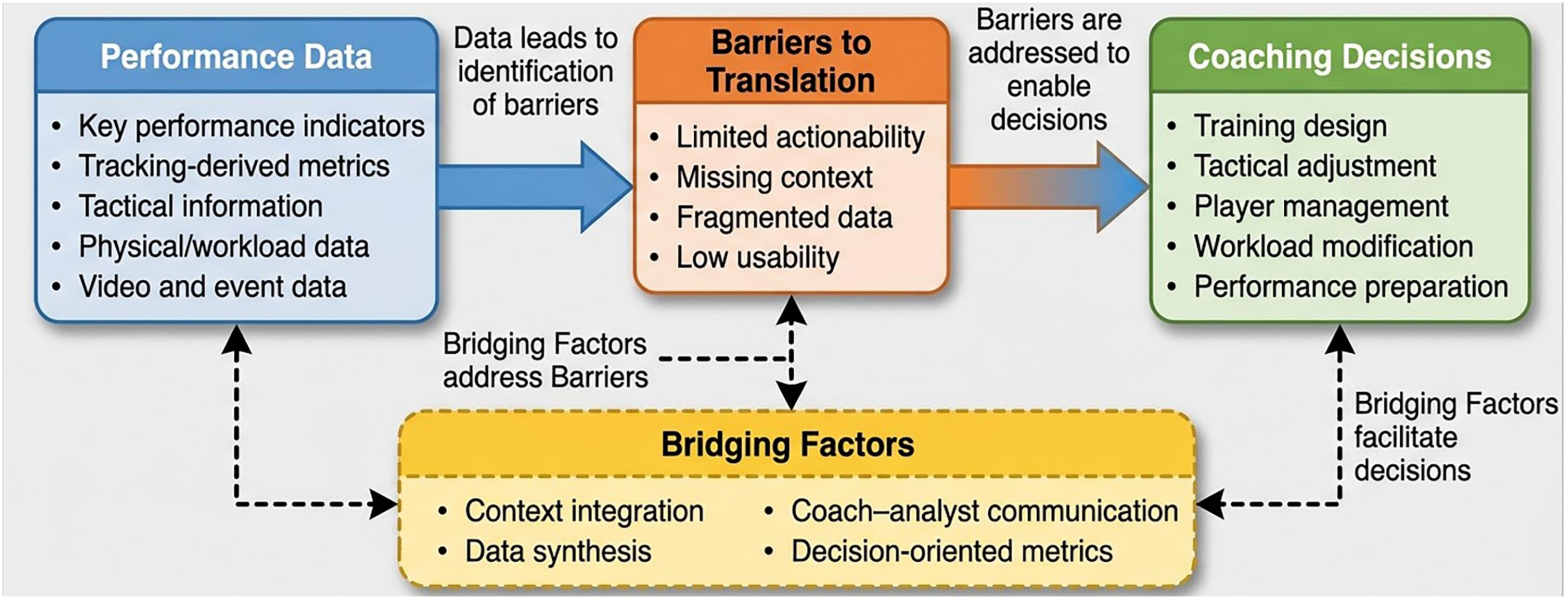
Decision-first framework for bridging the analytics–practice gap in sports performance. The figure illustrates how performance data, including key performance indicators, tracking-derived metrics, tactical information, physical/workload data, and video/event data, may fail to inform coaching decisions when analytical outputs lack actionability, contextual interpretation, integration, or usability. Bridging factors reduce these barriers by supporting context integration, data synthesis, coach–analyst communication, and decision-oriented metrics that connect analysis to training design, tactical adjustment, player management, workload modification, and performance preparation.

These observations suggest that the analytics–practice gap is not simply a matter of incomplete implementation. It reflects a more basic problem in the relationship between data, interpretation, and decision-making in sport. From a practitioner perspective, bridging this gap requires more than improved analytical methods. Coaches, performance analysts, sport scientists, and medical or performance staff need a shared way to identify where data fail to inform action, discuss why that failure occurs, and decide what kind of response is possible within the coaching environment. This places the framework within routine practice: post-match review, pre-training planning, opposition preparation, workload discussion, and player-management decisions. Performance analysis has been used to examine decision-making in team sports, including decision accuracy, available options, defensive pressure, match context, and execution, which supports the view that analysis can move beyond description toward decision-relevant interpretation (15). Research on video feedback as a facet of performance analysis also indicates that delivery, feedback environment, scheduling, and collaboration influence whether analysis supports learning and development (16). This means that analysis should be understood as part of a working process, not as a report alone. This relationship is illustrated in Figure 1.

## 3 Why the gap exists

### 3.1 Metrics without actionability

One reason for the analytics–practice gap lies in how performance metrics are constructed and interpreted. Indicators are often designed to summarize what has occurred, rather than to guide what should happen next. This issue is not limited to simple statistics. It also concerns the way analytical outputs are linked, or not linked, to coaching decisions.

A distinction is needed between performance analysis, KPI-based reporting, and sports analytics. Performance analysis has traditionally involved the systematic observation, coding, and interpretation of sport performance (4). Within this tradition, KPIs such as possession, passing accuracy, shots on target, entries into attacking areas, or defensive actions are widely used to describe observable events (9, 17). These indicators can be useful, but they should not be treated as synonymous with sports analytics. In the present paper, sports analytics refers more specifically to statistical, computational, model-based, or spatiotemporal approaches that seek to identify patterns, estimate probabilities, classify performance profiles, or support decisions.

This distinction matters because descriptive indicators and analytical models can both fail to be actionable. A KPI may show that a team completed fewer forward passes than expected, but it may not explain whether the issue was poor spacing, opponent pressure, limited movement between lines, or a deliberate tactical choice. Similarly, a more advanced metric may detect a tactical pattern without clarifying how the coach should respond in training. Morgulev and Lebed argued that performance analysis and sports analytics often prioritize variables that are easily counted and widely accessible (6), rather than variables that explain the dynamic interactions of performance. Their critique is relevant here because the problem is not simply whether a metric is basic or advanced. The problem is whether the metric helps explain performance in a way that can inform a decision.

Sports analytics methods can include expected-goals models, possession-value models, passing-network analysis, tracking-derived measures of team spread or synchronization, entropy-based measures of movement or ball circulation, clustering of playing styles, data-driven opponent profiling, and predictive workload models. These approaches may account for probability, space, time, interaction, and patterning more directly than isolated KPIs, but they still require coaching interpretation. For example, low shot quality in an expected-goals model may reflect shot selection, chance creation, attacking spacing, opposition pressure, or the timing of final-third actions.

Actionability also depends on who defines the variables that are expected to guide action. In applied settings, these variables should not be selected by analysts alone. Coaches, analysts, sport scientists, and, where relevant, medical or performance staff may each see a different part of the same performance problem. For this reason, action variables should be defined in relation to the coaching problem, the team's playing model, and the intervention that is realistically available. A variable linked to pressing effectiveness, for example, may need to reflect the coach's intended pressing triggers, the analyst's interpretation of spacing and opposition behavior, and the physical capacity required to repeat the action. Its validity is therefore not only statistical. It is also practical and contextual. A useful action variable should represent the intended behavior, remain meaningful under the relevant match or training conditions, and point toward a feasible coaching response. Performance analysis has been used to examine decision-making in team sports through information such as available options, defensive pressure, match context, and execution quality, which supports the use of decision-relevant variables rather than isolated indicators (15). Recent applied work on a performance analysis framework for netball umpires also shows how performance variables can be made more usable when they are structured through a notation scheme, validated by domain experts, and connected to video examples within an applied learning process (18).

Herold et al. provide an applied example from professional football (12). Their tracking-derived measures of defensive disruption and number of outplayed opponents were intended to capture how passes affected defensive organization and opponent elimination, rather than simply count events. However, when these metrics were used in a video-feedback intervention, they did not produce significant improvements in the main tracking-derived outcomes. This illustrates the issue of actionability: even analytically meaningful metrics may have limited practical impact if they are not connected to coaching explanation, training task design, and usable player feedback.

Metrics without actionability therefore create a weak link between analysis and practice. They may describe performance, compare players or teams, or identify patterns, but still fail to answer the practical question facing the coach. The relevant question is not only “What does the metric show?” but also “What decision should this information support?” Without this connection, performance analysis and sports analytics risk producing information that is accurate, detailed, and professionally presented, but only loosely connected to coaching action.

### 3.2 The absence of context

A second issue concerns context. In applied coaching environments, context is not only a theoretical concept. It includes practical factors such as opponent quality, match status, playing style, player availability, fixture congestion, training phase, tactical objectives, and the time remaining before the next match. These factors shape both what data should be collected and how that data should be interpreted. A sprint-distance value, for example, may carry different meanings depending on whether it occurred during a congested fixture period, in a match where the team defended deep, or in a week when the tactical plan required repeated high pressing.

This means that performance should not always be captured in the same way across time. Week to week, analysis may need to focus on immediate tactical problems, opposition-specific preparation, player readiness, or recovery demands. Across a month, the emphasis may shift toward trends in workload, tactical consistency, adaptation to training, or recurring performance issues. Across a season, the same data may be used to examine player development, injury risk, changes in playing style, or whether the team's performance model remains stable under different competitive conditions. Context therefore affects not only interpretation, but also the selection of metrics and the timing of analysis.

Match analysis research supports this point. The meaning of technical actions depends on variables such as match status and opposition level (11). More recent umbrella-review evidence also indicates that match context, including match status, match location, match period, and quality of opposition, requires deeper consideration in team ball sport research (8). The same metric can therefore reflect different realities depending on the situation. High possession may indicate territorial control in one match, sterile circulation in another, or a deliberate response to protecting a lead.

Ecological approaches remain useful because they explain performance behavior as emerging from interactions among players, tasks, opponents, and environmental constraints. From this perspective, a metric should not be interpreted as an isolated output. It is a behavior shaped by tactical intentions, available affordances, and the constraints present in a specific performance environment (19). This view also connects with ecological accounts of skill acquisition, where learning and performance are shaped by the interaction between the performer and the surrounding task environment (20). The same physical or technical value may therefore support different coaching interpretations across matches, training phases, or opposition contexts. Representative learning design extends this logic into practice by emphasizing that training tasks should preserve key information–movement relations from competition, rather than separate behavior from the conditions in which it occurs (21). In applied coaching environments, however, these theoretical ideas still need to be translated into operational questions: what was the team trying to do, what did the opponent allow or prevent, what constraints shaped the behavior, and what decision should follow?

### 3.3 Fragmented data and isolated perspectives

A third issue is the fragmented organization of performance data. In many applied environments, technical, tactical, physical, and medical information are collected through different systems and interpreted by different staff members. Event data may describe passes, shots, turnovers, and defensive actions. Tracking data may describe spacing, speed, acceleration, and team shape. Wearable sensors may describe external load, while wellness or medical information may provide insight into fatigue, soreness, or readiness. Each source can be useful, but coaching decisions often require these sources to be interpreted together rather than separately.

This fragmentation can lead to incomplete or misleading interpretations. Reduced sprint distance may appear to be a physical issue if viewed only through workload data, but it may also reflect tactical role, defensive block height, available space, or deliberate energy management. Similarly, lower passing accuracy may suggest technical underperformance, but may also reflect opponent pressure, build-up structure, or a tactical choice to play more progressive passes. In both cases, interpretation depends on the relationship between physical output, tactical context, technical execution, and coaching intention.

This issue becomes especially important when performance is examined across different time scales. A week-to-week decline in high-intensity running may require immediate attention if it coincides with fatigue, fixture congestion, or reduced recovery. Across a month, the same pattern may be more relevant to training adaptation, tactical consistency, or changing positional demands. Across a season, it may indicate a shift in playing style, player development, or cumulative workload risk. The same variable can therefore carry different practical meanings depending on whether it is being used for short-term preparation, medium-term monitoring, or long-term performance planning.

Research on performance and match analysis supports the need for more integrated interpretation, particularly when analysis aims to explain performance rather than only describe isolated events (22). Sarmento et al. argued that team ball sport research needs stronger integration of physical, technical, tactical, and psychosocial dimensions to better explain performance and bridge the gap between theory and practice (8). This point is directly relevant to applied analytics. A coach rarely needs to know only whether a player ran less, passed worse, or occupied a different space. The more useful question is why this occurred, whether it matters for the next decision, and what should be changed in training or preparation.

Morgulev and Lebed also emphasize that performance in team invasion sports emerges through dynamic interactions, rather than from isolated variables (6). From this perspective, technical actions, tactical positioning, physical demands, and contextual constraints should not be treated as independent pieces of information. These dimensions interact. A pressing strategy changes running demands. Opponent pressure changes passing options. Player fatigue changes movement timing and decision quality. Tactical role changes the physical meaning of the same workload output.

For analytics to become more useful in practice, integration should therefore be decision-oriented rather than merely descriptive. Combining multiple data sources is not valuable simply because it creates a larger dataset. It is valuable when it helps clarify a coaching problem. For example, if a team struggles to progress the ball under pressure, useful analysis may need to combine passing networks, player spacing, opponent pressing behavior, ball-loss locations, and physical capacity to repeat high-intensity movements. If a player's workload has increased, interpretation may need to include tactical role, match exposure, recovery status, injury history, and upcoming fixtures. Integration should help coaches move from separate observations toward a coherent explanation.

Fragmented data therefore contribute to the analytics–practice gap by preventing coaches and analysts from seeing how different dimensions of performance interact. The practical task is not simply to collect technical, tactical, physical, and contextual data, but to synthesize them around the decisions that matter. Without that synthesis, analysis may remain accurate within each domain while still failing to support coaching action.

### 3.4 Practical constraints in coaching environments

Practical constraints also limit the translation of analytics into coaching practice. Even when relevant data are available, coaches and support staff may not have the time, resources, or shared understanding needed to turn those data into usable decisions. This issue is especially important in applied environments, where decisions are often made under time pressure and where the next training session or match may arrive before a detailed analysis can be completed.

These constraints are not only logistical. They also concern how knowledge is delivered, interpreted, and acted upon within the performance environment. Bartlett and Drust argued that effective performance delivery in professional sport depends on translating scientific knowledge into forms that fit the needs, timing, and working realities of practitioners (23). This matters for analytics because a technically sound output may still have little practical value if staff do not have time to interpret it, if its meaning is unclear, or if it is not connected to the decision being made. Performance analysis practice also depends on the relationship between coaches and analysts. Nicholls et al. showed that coaches and analysts may experience performance analysis differently, which reinforces the need for shared expectations, clear communication, and an agreed understanding of how information will be used (24).

Manpower is one obvious constraint. In some high-performance settings, analysts, sport scientists, medical staff, and coaches may each contribute to the interpretation of performance data. In other environments, however, one or two staff members may be responsible for coding video, preparing reports, monitoring workload, communicating with coaches, and supporting training design. Under these conditions, the problem is not only whether data exist. It is whether there is enough staff capacity to interpret the data, discuss their meaning, and translate them into coaching action.

Financial resources create a similar problem. Advanced tracking systems, wearable sensors, video platforms, data providers, software licenses, and technical support can be expensive. Even when these tools are available, their value depends on whether staff have the time and expertise to use them properly. A club may be able to purchase a platform, but this does not guarantee that the platform will improve training design or tactical preparation. Without a clear decision-making purpose, investment in analytics can increase the amount of information available without improving its practical use.

Coach understanding is another important factor. This does not mean that coaches need to become data scientists. Rather, they need to understand what an output means, what it does not mean, and how much confidence should be placed in it. For example, a coach may not need to know the full statistical structure of a predictive workload model, but still needs to understand whether the model is identifying a meaningful risk, a normal fluctuation, or a signal that requires discussion with medical and performance staff. This is particularly relevant to workload decisions, where the relationship between training load, adaptation, and injury risk is complex rather than simply linear (25). If the meaning of the output is unclear, coaches are likely to rely on familiar information, experience, or simpler indicators that can be acted on quickly.

This also raises a more basic question: is more analytics always needed? In many cases, the answer is no. Additional analysis is useful only when it improves a decision that matters. If a metric does not affect training design, tactical planning, player management, communication, or performance preparation, then its practical value is limited. The issue is not whether a club can collect more data, but whether the analysis helps answer a coaching problem that could not be answered as well through existing knowledge, observation, or discussion.

Usability is therefore central. Coaches operate under time constraints and often need information that can be interpreted quickly. Research on athlete monitoring suggests that practitioners often value measures that are simple and interpretable, particularly when they need to support applied decisions (26). This does not mean that complex analytics should be avoided. It means that complex analysis must be translated into clear implications, with uncertainty, assumptions, and practical limitations communicated in a way that coaches can use.

Finally, the usefulness of analytics depends on how well it aligns with training practice. Skill acquisition research emphasizes the importance of representative training environments (27). Data that remain separate from training design are therefore less likely to influence performance. For analytics to matter, its outputs should help shape training constraints, feedback, player roles, tactical scenarios, or preparation strategies. Otherwise, analysis may remain informative in a meeting or report, but disconnected from the actual process through which performance is developed.

## 4 From analysis to action: a decision-first framework

Moving from analysis to action requires a change in the starting point of the analytical process. The term decision-first is used here as an author-proposed organizing principle, rather than as an established named framework. It is intended to synthesize relevant work on performance analysis, decision-making, and professional practice in applied sport settings. A common theme across this literature is that information becomes more useful when it is connected to the decision, context, and intervention it is meant to support. Performance analysis has been used to examine decision-making in team sports, including how players respond to available options, defensive pressure, match context, and execution demands (15). Applied performance analysis research also shows that professional practice depends on contextual awareness, technical expertise, relationships, and professional behaviors, rather than on data production alone (28). In this paper, decision-first analytics therefore refers to a practical way of organizing analysis around the coaching decision before selecting metrics, interpreting outputs, or designing feedback.

A decision-first approach begins with the coaching problem. This problem may be tactical, such as how to adjust pressing behavior against a specific opponent. It may relate to training design, such as how to create constraints that improve ball progression under pressure. It may concern selection, player management, recovery, or workload modification during a congested fixture period. Starting with the decision helps determine which data are needed, which metrics are relevant, and which forms of interpretation are likely to be useful. It also prevents analysis from becoming an exercise in reporting everything that is available.

This does not mean that complex data should be avoided. Advanced monitoring and analysis systems can provide valuable information about technical actions, tactical patterns, physical demands, and player responses (2, 13). However, their value depends on whether they are connected to a specific coaching purpose. If the decision concerns tactical preparation, isolated event counts may be insufficient. Video, event data, tracking-derived spacing information, passing networks, and opponent behavior may need to be interpreted together. If the decision concerns workload management, external-load data may need to be considered alongside match exposure, training history, recovery status, injury history, and tactical role. The aim is not to include every available variable, but to identify the information that clarifies the coaching problem.

Context is central to this process. The same analytical output can imply different coaching responses depending on match status, opposition level, player availability, fixture congestion, and tactical intention. A reduction in high-intensity running, for example, may indicate fatigue in one situation, a deeper defensive strategy in another, or reduced opportunity to run in behind the opposition. Sarmento et al. emphasized the need for more integrated match analysis that considers physical, technical, tactical, and contextual dimensions of performance (8). This is important because coaching decisions are rarely based on one dimension of performance alone.

Communication is also part of translation. Coaches do not only need accurate information; they need information that can be understood quickly and linked to intervention. This may involve short video clips connected to specific metrics, visual summaries that show where a tactical problem occurs, or brief decision notes that outline possible coaching responses. Research on athlete monitoring has similarly suggested that practitioners often value measures that are simple and interpretable when they are used to support applied decisions (26). The point is not to remove complexity from the analysis, but to communicate its practical meaning clearly enough for coaches to use it.

For analysis to influence performance, it must eventually connect to something that can be changed. This may include training constraints, tactical scenarios, feedback language, player roles, recovery plans, or match preparation. Skill acquisition research has emphasized the importance of representative training environments, where practice conditions reflect the demands of performance (27). This is relevant to analytics because data are unlikely to change performance if they remain separate from the training environment. A tactical problem identified through analysis should therefore be translated into practice tasks, player feedback, and coaching interventions that target the behavior in question.

This point is reinforced by intervention work using tracking-derived football metrics, where analytically meaningful feedback did not necessarily produce measurable improvements unless it was connected to coaching explanation, field-based practice, and behavioral change.

A decision-first framework should therefore include evaluation. Once analysis has been translated into training or preparation, coaches and analysts need to ask whether the intended behavior changed. This evaluation does not always need to be complex. It may involve comparing video examples before and after a training block, examining whether a tactical behavior appears more consistently across matches, or checking whether workload responses stabilize after an intervention. What matters is that analysis returns to the original coaching problem. The process should not end with a report; it should ask whether the action taken was useful.

The decision-first framework proposed here can therefore be understood as a practical cycle: define the coaching decision, select relevant information, interpret it in context, communicate its practical meaning, translate it into intervention, and evaluate whether the intervention changed the intended behavior. This framework does not reject advanced analytics. Rather, it positions analytics within the realities of coaching practice. Figure 1 summarizes this logic by showing why performance data may fail to inform coaching decisions when barriers such as limited actionability, weak contextual interpretation, fragmented information, or low usability are not addressed. It also shows how bridging factors, such as context integration, data synthesis, coach–analyst communication, and decision-oriented metrics, can reduce these barriers. The purpose of the framework is therefore not to add another layer of reporting, but to make the pathway from information to coaching action more explicit. Complex models, tracking-derived metrics, and integrated datasets are most valuable when they help coaches decide what to adjust, when to adjust it, and how to judge whether the adjustment worked.

## 5 Embedding the framework in coaching practice

Embedding the decision-first framework in coaching practice requires clarity about who uses it and when it is used. The framework is intended for shared use by coaches, performance analysts, sport scientists, and medical or performance staff. Coaches are central because they define the coaching problem and decide whether the response should involve training design, tactical adjustment, selection, feedback, recovery, or player management. Analysts then help select and interpret relevant video, indicators, tracking-derived information, or model outputs. Sport scientists and medical staff may add information about workload, readiness, recovery, and injury risk when these factors shape the decision.

In practice, the framework may be used during post-match review, pre-training planning, opposition preparation, workload meetings, or player-management discussions. It is not intended to replace existing coaching routines. Rather, it gives those routines a clearer decision structure. Video-based performance analysis already plays an important role in coach feedback and applied review processes, particularly when coaches use analysis to shape what players see, discuss, and practise next (5). Coach–analyst work also depends on shared expectations and communication. Coaches and analysts may not always experience the purpose or use of performance analysis in the same way (24). For this reason, the framework should be treated as a practical conversation tool as much as an analytical model.

Analytics should also be linked directly to the design of practice tasks. If analysis shows that a team struggles to progress the ball under pressure, the next step is not simply to present this information to players. The information should help shape training constraints, pitch dimensions, player numbers, starting positions, scoring rules, or feedback cues. In this way, analysis becomes part of the learning environment rather than a separate description of past performance. This is consistent with the broader principle that practice tasks should represent the demands of performance, rather than isolate behaviors from the context in which they occur (27).

Communication between coaches, analysts, sport scientists, and medical staff is also central. Many applied decisions require more than one perspective. A player's reduced high-intensity running may be a physical issue, a tactical consequence, a readiness concern, or a planned response to match demands. For this reason, analytics should be discussed in short, decision-oriented conversations rather than only delivered through detailed reports. The aim is not to simplify performance excessively, but to make the interpretation usable for the people who must act on it.

This also changes the role of the analyst. The analyst is not only a producer of information, but also a translator between data and coaching action. This role requires an understanding of the team's playing model, training priorities, coach preferences, and practical constraints. It also requires the ability to communicate uncertainty. A metric may indicate a possible problem, but coaches still need to know whether the signal is strong enough to justify a change, whether alternative explanations exist, and what the consequences of acting on the information might be.

For coaches, the framework suggests that analytics should be used selectively. More data are not always needed. Additional analysis is worthwhile when it helps clarify a decision that affects training design, tactical planning, player management, recovery, or match preparation. If a metric does not change what coaches do, how they communicate, or how training is organized, its practical value should be questioned. The purpose of analytics is not to replace coaching judgement, but to sharpen it by making relevant performance problems more visible and discussable.

For training design, the framework encourages a cycle of analysis, intervention, and evaluation. A performance problem is first identified in relation to a coaching decision. Relevant data are then interpreted in context and translated into a training intervention. After the intervention, coaches and analysts examine whether the intended behavior changed. This may involve video comparison, tracking-derived behaviors, tactical indicators, player feedback, or workload responses. The evaluation does not need to be overly complex, but it should return to the original decision. Without this step, it is difficult to know whether analysis actually influenced performance.

These implications also show why analytics must be adapted to the resources of the environment. In a well-resourced professional club, the framework may involve integrated data platforms, tracking-derived models, interdisciplinary meetings, and regular intervention reviews. In a smaller program, it may involve a simpler process: one coaching question, one or two relevant indicators, selected video clips, and a short review after training or competition. The principle remains the same. Analytics should be scaled to the setting, but still connected to decisions, practice design, and evaluation.

Embedding analytics in practice therefore requires more than improved technology. It requires routines that connect data to coaching problems, interpretation to training design, and feedback to behavioral change. The decision-first framework provides one way to structure this process. Its value lies in making the pathway from analysis to action explicit, so that performance data are not only collected and reported, but used to support coaching decisions that can be tested in training and competition.

## 6 Conclusion: closing the gap

The expansion of performance data in sport has created new possibilities for understanding training and competition. Tracking systems, wearable sensors, video platforms, event data, and analytical models can describe performance in considerable detail, but the central issue is whether these data help coaches make better decisions.

This paper has argued that the analytics–practice gap is shaped by four connected problems: metrics that describe performance without guiding action, insufficient contextual interpretation, fragmented data sources, and practical constraints within coaching environments. Closing this gap requires a shift from data-first to decision-first analytics, in which analysis begins with the coaching decision, selects relevant information, interprets it in context, communicates it in usable forms, translates it into intervention, and evaluates whether the intended behavior changed. The practical value of sports analytics therefore depends less on the amount of information collected than on the quality of the connection between information, coaching action, and performance development.
